# Feature Selection Based on a Large-Scale Many-Objective Evolutionary Algorithm

**DOI:** 10.1155/2021/9961727

**Published:** 2021-08-24

**Authors:** Yue Li, Zhiheng Sun, Xin Liu, Wei-Tung Chen, Der-Juinn Horng, Kuei-Kuei Lai

**Affiliations:** ^1^State Key Laboratory of Reliability and Intelligence of Electrical Equipment, Hebei University of Technology, Tianjin, China; ^2^School of Economics and Management, Hebei University of Technology, Tianjin, China; ^3^Department of Business Administration, NCU, Taoyuan, China; ^4^Department of Business Administration of Chaoyang University of Technology, Taichung, China

## Abstract

The feature selection problem is a fundamental issue in many research fields. In this paper, the feature selection problem is regarded as an optimization problem and addressed by utilizing a large-scale many-objective evolutionary algorithm. Considering the number of selected features, accuracy, relevance, redundancy, interclass distance, and intraclass distance, a large-scale many-objective feature selection model is constructed. It is difficult to optimize the large-scale many-objective feature selection optimization problem by using the traditional evolutionary algorithms. Therefore, this paper proposes a modified vector angle-based large-scale many-objective evolutionary algorithm (MALSMEA). The proposed algorithm uses polynomial mutation based on variable grouping instead of naive polynomial mutation to improve the efficiency of solving large-scale problems. And a novel worst-case solution replacement strategy using shift-based density estimation is used to replace the poor solution of two individuals with similar search directions to enhance convergence. The experimental results show that MALSMEA is competitive and can effectively optimize the proposed model.

## 1. Introduction

Feature selection involves the selection of a specific number of features from existing features to optimize specific objectives [1]. Feature selection can be regarded as a multiobjective optimization problem that can be solved using evolutionary algorithms. Feature selection has attracted the attention of scholars and has been widely used in gene expression analysis [2], face recognition [3], and drug discovery [4]. For example, a two-stage heuristic algorithm minimal redundancy maximal relevance (mRMR) [5] is used to optimize relevance and redundancy simultaneously. A filter-based algorithm [6] is used to consider the entropy-based correlation measure and the combination measure of the redundancy and cardinality of a selected subset. A decomposition algorithm based on a weighted method is utilized to optimize interclass and intraclass distances [7]. Gulsah et al. [8] proposed two algorithms, W-QEISS and F-QEISS, that use nondominated sorting based on classification accuracy, feature number, relevance, and redundancy. Li et al. [9] established a model with feature number, classification performance, interclass distance, and intraclass distance as objectives and proposed a decomposition-based large-scale algorithm (DMEA-FS).

However, some unsolved problems still exist in feature selection using traditional evolutionary algorithms. The first problem is that the selection of a large number of features can be regarded as the optimization of the large-scale optimization problem [1] or the large-scale multiobjective optimization problem (LSMOP) [10], but the traditional evolutionary algorithms cannot effectively solve such problems. The second problem is that feature number and accuracy are two basic objectives, and other objectives are needed to explore the potential information to guide the evolution in feature selection [1]. Correspondingly, more objectives result in many-objective optimization problems (MaOPs) [11, 12].

There are three main types of current algorithms, which are mainly used to solve LSMOPs or MaOPs, but they perform poorly on large-scale many-objective problems (LSMaOPs) [13], which include more than 3 objectives and over 100 decision variables [14, 15].

The first kind of algorithms is based on the Pareto dominance, which improves the convergence pressure by modifying the Pareto dominance relation. The new dominance relations are *ε*-dominance [16], *θ*-dominance [17], *L*-optimality [18], simplex dominance [19], grid dominance [20, 21], etc. The algorithm using shift-based density estimation (SDE) was proposed in the work of [22], which allows individuals with poor convergence to obtain higher density.

The second is based on performance indicators, such as the hypervolume (HV) adaptive grid algorithm (HAGA) [23], the evolutionary algorithm (MaOEA/IGD) using inverted generational distance (IGD) [24], indicator-based algorithm with boundary protection (MaOEA-IBP) [25], and R2 indicator and weight vector-based method (R2-WVEA) [26]. Most of these algorithms are many-objective evolutionary algorithms (MaOEAs), but their computational costs are large.

The third category is composed of decomposition-based methods. The most classic ones are the multiobjective evolutionary algorithm based on decomposition (MOEA/D) [27] and its variants [28–30]. The algorithm based on nondominated sorting approach (NSGA-III) [31] uses evenly distributed reference points to assist the environmental selection. Based on NSGA-III, Gu and Wang [10] introduced an information feedback model to solve LSMaOPs. The reference vector-guided evolutionary algorithm (RVEA) [32] uses reference vectors to guide the optimization.

To more comprehensively describe and better solve the large-scale feature selection problem, this paper studies the existing multiobjective models based on the evolutionary algorithm, combines the existing objectives, constructs the feature selection problem as an LSMaOP, and uses an improved large-scale many-objective evolutionary algorithm (LSMaOEA) for optimization.

The main contributions of this paper are summarized as follows:

1. A novel worst-case solution replacement strategy based on SDE is proposed. This strategy allows conditional replacement of poor solutions in terms of convergence and diversity compared to other solutions, thereby maintaining a balance between convergence and diversity.
2. A modified vector angle-based large-scale many-objective evolutionary algorithm (MALSMEA) is proposed, which uses variable grouping-based polynomial mutation instead of naive polynomial mutation to improve the efficiency of solving large-scale problems. In the environmental selection process, the proposed worst solution replacement strategy is used to improve diversity.
3. A large-scale many-objective feature selection optimization model is constructed, and MALSMEA is used to optimize it. The optimization objectives of this model are the number of selected features, accuracy, relevance, redundancy, interclass distance, and intraclass distance.

The remainder of this paper is arranged as follows.  Section 2 introduces the related works.  Section 3 describes the proposed model and MALSMEA in detail. In Section 4, we compare and analyze the experimental results of MALSMEA and four advanced algorithms in solving benchmark LS-MaOPs, as well as the performance of MALSMEA and three feature selection algorithms in optimizing the proposed feature selection model.  Section 5 provides a summary of the full paper and prospects of future research.

## 2. Related Works

### 2.1. Large-Scale Many-Objective Optimization Problem

An LSMaOP can be described as

(1) $$\begin{array}{c} \min\;F\left(x\right)=\left(f_{1}\left(x\right),f_{2}\left(x\right),\ldots ,f_{m}\left(x\right)\right)\\ \text{s.t.}\;x\in \Omega , \end{array}$$

where Ω=∏_*i*=1_^*D*^[*l*_*i*_, *u*_*i*_]⊆*R*^*D*^ is the decision space, *D* is the number of decision variables (*D* ≥ 100), and *l*_*i*_ and *u*_*i*_ are the lower and upper bounds of decision variables in the *i*th dimension, respectively. *x* is the *D*-dimensional decision vector in Ω, *m* is the objective number (*m* > 3), and *F*(*x*) ∈ *R*^*m*^ is the objective vector of *x*. If no other solution dominates *x*, then *x* is a Pareto optimal solution [33]. The objective vectors corresponding to all Pareto optimal solutions constitute the Pareto optimal front (PF) [34, 35].

### 2.2. Shift-Based Density Estimation

We use the SDE [22] with the *k*th nearest neighbor [36] to estimate the density of all individuals. For an individual *x*_*i*_, the following method is used to calculate the density value SDE(*x*_*i*_).

(i) First, the standardized objective vectors of other individuals in population *P* are shifted.
(ii) Then, the Euclidean distances between other shifted normalized objective vectors and the considered individual are calculated, expressed as *d*(*x*_*i*_, *x*_*k*_).
(iii) Next, the *k*th minimum value *λ*(*x*_*i*_) in the set {*d*(*x*_*i*_, *x*_*k*_), *x*_*k*_ ∈ *P*∩*x*_*k*_ ≠ *x*_*i*_} is found, where $k=\sqrt{N}$ and *N* is the size of the population.
(iv) Finally, SDE(*x*_*i*_) is calculated as follows:
  (2) $$\begin{array}{c} \text{SDE}\left(x_{i}\right)=\frac{1}{\lambda \left(x_{i}\right)+2}. \end{array}$$

Through the above process of estimating the individual density, we can observe that the smaller the individual density is, the better the performance of the individual. Therefore, this paper uses this strategy, considering both diversity and convergence, to judge a pair of individuals with similar search direction, so as to delete the individual with poor performance.

### 2.3. Information Theory Criterion Based on Entropy

The feature selection model uses an entropy-based information theory criterion [8] to measure correlation and redundancy. For a given discrete random variable *A*, its entropy *E*(*A*) is determined as follows:

(3) $$\begin{array}{c} E\left(A\right)=-\sum_{a\in A}p\left(a\right)\log\;p\left(a\right), \end{array}$$

where *p*(*a*)=Pr(*A*=*a*), *A* is the set of all possible values of *A*, *a* ∈ *A*. Then, the joint entropy of *A* and *B* is determined as follows:

(4) $$\begin{array}{c} E\left(A,B\right)=-\sum_{a\in A}\sum_{b\in B}p\left(a,b\right)\log\;p\left(a,b\right), \end{array}$$

where *B* is a discrete random variable, *p*(*a*, *b*)=Pr(*A*=*a*, *B*=*b*), *a* ∈ *A*, and *b* ∈ *B*. Then, the mutual information between *A* and *B* is determined as follows:

(5) $$\begin{array}{c} M\left(A,B\right)=E\left(A\right)+E\left(B\right)-E\left(A,B\right). \end{array}$$

Symmetric uncertainty is used to scale the value range of mutual information to [0,1] [37], which is defined as follows:

(6) $$\begin{array}{c} \text{SU}\left(A,B\right)=\frac{2M\left(A,B\right)}{E\left(A\right)+E\left(B\right)}. \end{array}$$

## 3. Proposed Model and Algorithm

### 3.1. Model Design

The optimization objectives of the feature selection model include the number of selected features, accuracy, relevance, redundancy, interclass distance, and intraclass distance, which are described as follows:

(1) *The Number of Selected Features*. It is minimized to ensure the simplification of feature selection:
  (7) $$\begin{array}{c} F_{1}\left(S\right)=\left|S\right|, \end{array}$$
where |*S*| represents the cardinality of feature set *S*.
(2) *Accuracy.* The accuracy of the learning algorithm is measured by the classification performance. The higher the classification performance is, the greater the accuracy. In this paper, the extreme learning machine (ELM) classifier [8] is used to calculate the accuracy:
  (8) $$\begin{array}{c} F_{2}\left(S\right)=\frac{\text{tn}+\text{tp}}{\text{fn}+\text{fp}+\text{tn}+\text{tp}}, \end{array}$$
where tn, tp, fn, and fp represent the true negative, true positive, false negative, and false positive, respectively.
(3) *Relevance*. The relevance between features and categorical variables reflects the recognition ability of the selected features. The greater the correlation is, the stronger the recognition ability is:
  (9) $$\begin{array}{c} F_{3}\left(S\right)=\sum_{x_{i}\in S}\text{SU}\left(x_{i},y\right), \end{array}$$
where *x*_*i*_ represents the *i*th feature and *y* represents the target categorical variable. This objective is normalized according to *F*_3_(*S*)=*F*_3_(*S*)/max*F*_3_(*S*).
(4) *Redundancy*. The redundancy is used to quantify the level of similarity between selected features. The smaller the redundancy is, the smaller the similarity:
  (10) $$\begin{array}{c} F_{4}\left(S\right)=\sum_{x_{i},x_{j}\in S,i<j}\text{SU}\left(x_{i},x_{j}\right), \end{array}$$
where *x*_*j*_ represents the *j*th feature. This objective is normalized according to *F*_4_(*S*)=*F*_4_(*S*)/max*F*_4_(*S*).
(5) *Interclass Distance*. The interclass distance represents the distance between the mean sample of each class and the average of mean samples of all classes, which reflects the recognition ability of samples of different classes. In the evolutionary process, a better sample distribution is obtained by maximizing the distance between classes:
  (11) $$\begin{array}{c} F_{5}\left(S\right)=\sum_{i=1}^{L}{\left(m_{i}-\frac{1}{L}\sum_{i=1}^{L}m_{i}\right)}^{2}, \end{array}$$
where *L* is the total number of classes and *m*_*i*_ is the average value of all samples with feature *S* in class *i*. This objective is normalized according to *F*_5_(*S*)=*F*_5_(*S*)/max*F*_5_(*S*).
(6) *Intraclass Distance*. By calculating the distances between the samples with the selected feature and the mean of all samples of the same kind, this value reflects the cohesion of the same kind of samples and can improve the accuracy to a certain extent:
  (12) $$\begin{array}{c} F_{6}\left(S\right)=\sum_{i=1}^{L}\sum_{a_{ij}\in L_{i}}{\left(a_{ij}-m_{i}\right)}^{2}, \end{array}$$
where *a*_*ij*_ is the *j*th sample in class *i*. This objective is normalized according to *F*_6_(*S*)=*F*_6_(*S*)/max*F*_6_(*S*).

Therefore, the definition of the feature selection optimization model in this paper is as follows:

(13) $$\begin{array}{c} \min\left(F_{1}\left(S\right),-F_{2}\left(S\right),-F_{3}\left(S\right),F_{4}\left(S\right),-F_{5}\left(S\right),F_{6}\left(S\right)\right). \end{array}$$

### 3.2. The Proposed Algorithm: MALSMEA

**Figure 1:**
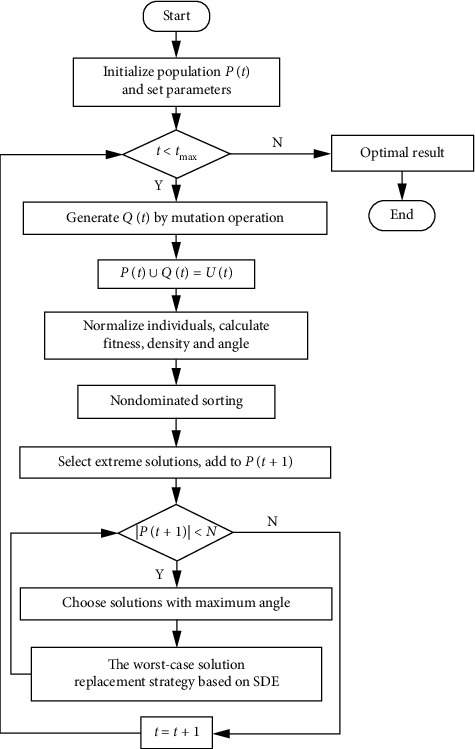
Program flowchart of MALSMEA.

In this paper, a modified vector angle-based large-scale many-objective evolutionary algorithm is proposed, termed as MALSMEA. MALSMEA mainly uses a mutation operator based on variable grouping and the environment selection method of VaEA [38].  Figure 1 shows the program flowchart of MALSMEA. The main process of MALSMEA is as follows:

- *Step 1*. Initialize a population *P*(*t*) with *N* individuals randomly in the whole decision space Ω, and set parameters.
- *Step 2*. The mutation operator based on variable grouping is used to mutate the population *P*(*t*), in which the grouping method is ordered grouping, to generate the offspring population *Q*(*t*).
- *Step 3*. Combine the offspring population *Q*(*t*) with the parent population *P*(*t*) and obtain the joint population *U*(*t*). Then, the environmental selection in steps 4–9 is adopted to select *N* promising individuals from *U*(*t*).
- *Step 4*. Normalize the individuals in the population *U*(*t*), and calculate the fitness and density values of each individual as well as the vector angle between every two individuals.
- *Step 5*. Use the nondominated sorting method to rank, and determine the last layer *F*(*l*).
- *Step 6*. According to the vector angle between any two individuals in layer *F*(*l*) and the fitness value of each individual, *m* individuals with the largest vector angle and *m* individuals with the smallest fitness value are selected to join *P*(*t*+1) to ensure the diversity.
- *Step 7*. If *|P*(*t*+1)*|* < *N*, select the individual with the largest vector angle in *F*(*l*) to join the new population *P*(*t*+1) by calculating the vector angles between the individuals in *F*(*l*) and the individuals in *P*(*t*+1); otherwise, go to step 9.
- *Step 8*. To maintain the balance between convergence and diversity, the worst individual replacement strategy is used to replace the poor individual with other individuals. Repeat from step 7 if |*P*(*t*+1)| < *N*.
- *Step 9*. Obtain the new population *P*(*t*+1).
- *Step 10*. Repeat from step 2, and stop when the maximum number of generations *t*_max_ is reached.

### 3.3. The Worst-Case Solution Replacement Strategy Based on SDE

As the extreme individuals have been selected according to the vector angle and fitness value, for the worst individual replacement strategy in the process of environmental selection, we use the SDE strategy to calculate the density of individuals. The SDE strategy can consider the convergence and diversity of individuals simultaneously. Using this method, we can replace the poor individuals with similar search directions. The specific process is as follows: if the angle between an individual *a* in *F*(*l*) and an individual *b* in *P*(*t*+1) is less than the angle between two solutions of *N* ideal solutions, that is, *θ*=((*π*/2)/*N*+1), where *N* is the population size, then they have similar search directions. In this case, if SDE(*a*) < SDE(*b*), then individual *b* is replaced by *a*. After replacement, the angle between each individual *a* ∈ *F*(*l*) and the new population *P*(*t*+1) is updated.

### 3.4. The Wrapper Structure of MALSMEA

MALSMEA is applied to the feature selection model, and the pseudocode of the wrapper structure of MALSMEA is shown in Algorithm 1. The main steps are as follows:

- First, the input dataset DS is divided into training and test datasets.
- Then, in the initialization process, MALSMEA allocates the random feature vector *W*_*S*_ selected from the data feature matrix *W*. The selected feature vector *W*_*S*_ is encoded as solutions by using the coding technology of [9] to reduce the amount of computation in the evolutionary process, and the mask of *W*_*S*_ is regarded as the decision variables, and the population *P* is formed.
- Then, in the wrapper structure, the population *P* is evaluated via six objective functions to obtain objective vectors and obtain the evaluated population *P*(*t*). The feature number is calculated according to the decision variables of the solutions. The accuracy can be obtained from the decoded feature subset and the corresponding ELM classifier [8], and other objectives can be calculated according to the corresponding equations.
- Then, the population is optimized by MALSMEA.
- Finally, the optimal set *P*_*S*_ is obtained.

### 3.5. Time Complexity Analysis

The time complexity of MALSMEA is composed mainly of the following parts: the time complexity of the mutation operation in MALSMEA is *O*(*D*^2^*N*/*K*), where *K* is the number of groups, the time complexity of nondominated sorting is *O*(*N*  log^*m*−2^  *N*) [31], the worst-case solution replacement strategy based on SDE has the time complexity of *O*(*mN*^2^), and the time complexity of other operations is *O*(*mN*^2^). Therefore, the time complexity of MALSMEA is max{*O*(*D*^2^*N*/*K*), *O*(*N*  log^*m*−2^  *N*), *O*(*mN*^2^)}. Compared with the four algorithms, the time complexity of the grouped and linked polynomial mutation operator (GLMO) is max{*O*(*D*^2^*N*/*K*), *O*(*mN*^2^)} [39], linear combination-based search algorithm (LCSA) is *O*(*mN*^2^) [40], vector angle-based evolutionary algorithm (VaEA) is max{*O*(*N*  log^*m*−2^  *N*), *O*(*mN*^2^)} [38], and RVEA is *O*(*mN*^2^) [32]. Thus, the time complexity of MALSMEA is similar to that of GLMO but greater than that of the other three algorithms.

## 4. Experimental Studies

In this section, DTLZ1-DTLZ6 in the Deb, Thiele, Laumanns, and Zitzler (DTLZ) test suite [41] and LSMOP1-LSMOP9 in the Large-Scale Multi- and Many-Objective Problems (LSMOP) test suite [42] are selected to evaluate the performance of MALSMEA, and four datasets in the University of California at Irvine (UCI) machine learning library [43] are selected to evaluate the ability of MALSMEA to optimize the proposed feature selection model, among which Heart is a two-class dataset, Zoo and Iris are two multiclass datasets, and Musk1 is a high-dimensional dataset. For LSMaOPs, MALSMEA is compared with GLMO [39], LCSA [40], VaEA [38], and RVEA [32]. GLMO and LCSA are large-scale multiobjective evolutionary algorithms. GLMO uses mutation operators based on variable grouping, and LCSA uses a linear combination to reduce dimensionality. VaEA and RVEA are many-objective evolutionary algorithms that use vector angles and reference vectors, respectively. For the proposed six-objective feature selection model, MALSMEA is compared with W-MOSS [44], W-QEISS, and F-QEISS [8].

In the next sections, we introduce the performance indicators and set the parameters in the experiments. Then, for all algorithms, when the objective numbers *m* are 5 and 10, the population sizes *N* are 126 and 275, and the numbers of decision variables *D* are 500 and 1000, respectively. Each algorithm runs 20 times independently and stops when the number of function evaluations (FEs) reaches 90,000. The performance of MALSMEA is verified by comparing the average IGD values obtained by five algorithms. In each test instance, the best average IGD value is highlighted in bold. Finally, in four datasets, MALSMEA and three feature selection algorithms are utilized to deal with the proposed six-objective feature selection optimization model, for which *N*=100, the maximum number of FEs is 100, and each algorithm runs independently for 10 times. The optimization ability of MALSMEA is verified by comparing the HV indicator and optimization results.

### 4.1. Experimental Settings

1. *Performance Indicator*. In the experiment, IGD [45] and HV [46] are used as evaluation indicators. The smaller (larger) the IGD (HV) indicator value is, the better the performance of the algorithm. The IGD indicator evaluates the algorithm by calculating the average of minimum distances between all sampled individuals on the actual PF and the obtained solution set. The HV indicator quantifies the algorithm performance by calculating the volume between the obtained nondominated solution set and the reference point.
2. *Parameter Settings for the Crossover and Mutation Operators*. In the performance verification experiment of MALSMEA, MALSMEA and GLMO use the mutation operator based on variable grouping to generate offspring. Other algorithms use simulated binary crossover (SBX) [32] and polynomial mutation [47]. The crossover probability is *p*_*c*_=1.0, the mutation probability is *p*_*m*_=1/*D*, and the distribution indicator is *η*_*m*_=20, where *D* is the number of decision variables. In the experiment to verify the superiority of MALSMEA with respect to the proposed model, according to [9], *p*_*c*_=0.8, *p*_*m*_=0.2.
3. *Other Parameter Settings for Algorithms*. In MALSMEA and GLMO [39], the number of groups *K* is set to 4, and the ordered grouping method is adopted. For RVEA [32], the index *α* and the frequency *f*_*r*_ are set to 2 and 0.1, respectively. The parameters in W-QEISS and F-QEISS are set according to [8], and the searching method is based on r-NSGA-II [48]. The parameters in W-MOSS are set according to [44].
4. *Datasets*. The details of 4 UCI datasets utilized are shown in Table 1.
5. *ELM Classifier*. For the proposed model, the ELM classifier [8] is utilized to evaluate the accuracy of the current solution, which follows the criterion given in [46]: the activation function is *g*(*x*)=1/(1+*e*^(−*x*)^) in the hidden layer, and the number of neurons is set to *n*_*h*_=10. The target classification variable and the (input) features are normalized into ranges [0, 1] and [−1, 1] in each dataset, respectively. To minimize the accuracy deviation, the *k*-fold cross validation approach is utilized with *k*=10, and the average accuracy is used for comparison [9].

### 4.2. Performance Comparison of Algorithms on DTLZ

Table 2 describes the IGD indicator values obtained by the five algorithms on the 5- and 10-objective DTLZ1-DTLZ6 with 500 and 1000 decision variables. As shown in Table 2, MALSMEA is competitive with the other four algorithms. Specifically, MALSMEA produces 18 best results out of 24 test instances, and its performance on the 10-objective DTLZ is significantly better than that of the other algorithms. The experimental results are analyzed in detail as below.

DTLZ1 reflects the convergence of the algorithm. MALSMEA outperforms the other algorithms on the 5- and 10-objective DTLZ1. These results demonstrate that MALSMEA has better convergence on the large-scale high-dimensional DTLZ1. DTLZ2 is generally used to test the scalability of algorithms with respect to the number of objectives. The performance of MALSMEA on the 5-objective DTLZ2 is better than that of LCSA but slightly inferior to that of GLMO, VaEA, and RVEA. The performance of MALSMEA on the 10-objective DTLZ2 is better than that of the other four algorithms. Thus, MALSMEA has better scalability to the objective number.

DTLZ3 is a highly multimodal problem similar to DTLZ1. MALSMEA obtains the smallest IGD indicator value on DTLZ3 with 500 and 1000 decision variables. DTLZ4 is used to test the ability of the algorithm to ensure the diversity of the population. MALSMEA obtains the smallest IGD indicator value on the 10-objective DTLZ4 with 500 and 1000 decision variables. For the 5-objective DTLZ4, VaEA outperforms other algorithms on DTLZ4 with 500 and 1000 decision variables. MALSMEA exhibits greater diversity on the large-scale 10-objective DTLZ4.

For the 5-objective DTLZ5, MALSMEA outperforms LCSA on DTLZ5 with 500 and 1000 decision variables, but inferior to GLMO, VaEA, and RVEA. For the 10-objective DTLZ5, MALSMEA outperforms its counterparts. For DTLZ6, the overall performance of MALSMEA is optimal on instances with up to 1000 decision variables.

To further test the performance of MALSMEA, the nonparametric Friedman test [49] is employed. According to the average IGD indicator values of the five algorithms on DTLZ, Table 3 indicates the average ranking of the five algorithms. The average ranking of MALSMEA is the smallest, which indicates that MALSMEA performs the best. The average ranking of LCSA is the largest, so its performance is the worst.

To verify the efficiency of MALSMEA, Table 4 presents the running time of MALSMEA and the four other algorithms on the 10-objective DTLZ1 with 1000 decision variables. The running times of MALSMEA and GLMO are quite similar but greater than those of other algorithms.

### 4.3. Performance Comparison of Algorithms on LSMOP

LSMOP is proposed to test the performance of the algorithm in LSMaOPs.  Table 5 lists the IGD indicator values obtained by five algorithms on 5- and 10-objective LSMOP1-LSMOP9 with 500 and 1000 decision variables. MALSMEA produces 26 best results out of 36 test instances. Therefore, compared with the other four algorithms, MALSMEA has better performance in solving LSMaOPs.

Specifically, for the LSMOP test suite with 500 decision variables, MALSMEA outperforms the other algorithms on the 5- and 10-objective LSMOP2, LSMOP4, LSMOP5, LSMOP8, and LSMOP9. MALSMEA is inferior to LCSA on LSMOP3. MALSMEA outperforms the other algorithms on the 10-objective LSMOP1 and LSMOP7, but LCSA obtains the smallest IGD indicator value on the 5-objective LSMOP1 and LSMOP7. MALSMEA obtains the smallest IGD indicator value on the 5-objective LSMOP6, while RVEA performs better on the 10-objective LSMOP6.

For the LSMOP test suite with 1000 decision variables, MALSMEA outperforms the other algorithms on the 5- and 10-objective LSMOP2, LSMOP4, LSMOP5, LSMOP8, and LSMOP9. MALSMEA is inferior to LCSA on LSMOP3. LCSA obtains the best performance on the 5-objective LSMOP1 and LSMOP7, and MALSMEA outperforms the other algorithms on the 10-objective LSMOP1 and LSMOP7. The performance of MALSMEA on the 5-objective LSMOP6 is better than that of the other algorithms, but it is slightly inferior to that of LCSA and RVEA on the 10-objective LSMOP6.

### 4.4. Comparison of the Optimization Results on the Proposed Model

Table 6 shows the HV indicator values and objective values of the four algorithms after optimization on four datasets. The results demonstrate that MALSMEA obtains the maximum HV indicator values, showing that MALSMEA has certain advantages in feature selection. As noted in Table 6, for the four datasets, the optimization performance of MALSMEA is better on Iris and Musk1. MALSMEA is slightly inferior to the other three algorithms in relevance and redundancy but exhibits better performance in the other four objectives. In addition, W-QEISS and F-QEISS are relatively better than the other algorithms in terms of relevance and redundancy, but they are worse in other objectives.

## 5. Conclusion

In this paper, a modified vector angle-based large-scale many-objective evolutionary algorithm called MALSMEA is proposed. In MALSMEA, the polynomial mutation based on variable grouping is used to replace the polynomial mutation to improve the efficiency of solving large-scale optimization problems. A novel worst-case solution replacement strategy based on SDE is proposed to replace the worse one of two individuals with similar search directions to increase diversity. In addition, MALSMEA is compared with four typical algorithms to solve the optimization problem with up to 10 objectives and 1000 decision variables. Experimental results indicate that MALSMEA outperforms the four algorithms on the DTLZ and LSMOP test suites. By studying the existing feature selection models, taking the number of selected features, accuracy, relevance, redundancy, interclass distance, and intraclass distance as the optimization objectives, a six-objective optimization model is constructed and solved by using MALSMEA. Compared with the other three feature selection algorithms, MALSMEA has some advantages in solving this model.

Future studies will proceed in two directions. The first direction is to add a parallel strategy to MALSMEA to improve efficiency or to further modify its environmental selection method. Another research direction is to solve LSMaOPs in other fields using MALSMEA.

## Figures and Tables

**Algorithm 1:**
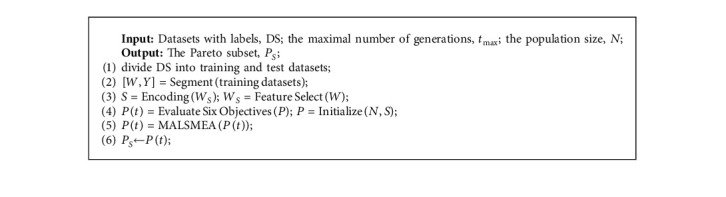
The wrapper structure of MALSMEA.

**Table 1:** The information of four UCI datasets.

Dataset	Classes	Features	Instance
Heart	2	13	270
Zoo	7	16	101
Iris	3	4	150
Musk1	2	166	476

**Table 2:** Performance comparison between MALSMEA and four algorithms with respect to the average IGD values on the DTLZ1-DTLZ6 (gray values represent the best values in each row).

Problem	*m*	*D*	MALSMEA	GLMO	LCSA	VaEA	RVEA
DTLZ1	5	500	1.1079*e* + 3 (4.24*e* + 2)	9.9478*e* + 3 (1.61*e* + 3)	3.9526*e* + 3 (2.52*e* + 2)	4.5327*e* + 3 (2.97*e* + 2)	7.8347*e* + 3 (1.99*e* + 2)
		1000	3.6284*e* + 3 (1.03*e* + 3)	1.8810*e* + 4 (2.97*e* + 3)	7.7836*e* + 3 (4.34*e* + 2)	1.3520*e* + 4 (5.87*e* + 2)	1.8532*e* + 4 (3.84*e* + 2)
	10	500	2.2202*e* + 3 (3.47*e* + 2)	9.4305*e* + 3 (5.82*e* + 2)	4.5825*e* + 3 (3.67*e* + 2)	8.4640*e* + 3 (3.85*e* + 2)	7.2316*e* + 3 (7.61*e* + 2)
		1000	4.7828*e* + 3 (9.45*e* + 2)	1.8648*e* + 4 (9.68*e* + 2)	9.2419*e* + 3 (4.31*e* + 2)	1.8151*e* + 4 (5.38*e* + 2)	1.6042*e* + 4 (3.96*e* + 2)

DTLZ2	5	500	2.8988*e* + 1 (1.27*e* + 0)	3.0185*e* + 1 (4.19*e* + 0)	2.9554*e* + 1 (2.79*e* + 0)	4.0643*e* + 0 (2.88*e* - 1)	2.5720*e* + 0 (1.80*e* − 1)
		1000	6.6661*e* + 1 (2.14*e* + 0)	6.2634*e* + 1 (6.02*e* + 0)	7.7019*e* + 1 (5.98*e* + 0)	1.8169*e* + 1 (8.34*e* − 1)	1.4208*e* + 1 (6.65*e* − 1)
	10	500	2.1635*e* + 1 (3.68*e* + 0)	3.7764*e* + 1 (4.64*e* + 0)	4.2687*e* + 1 (1.30*e* + 1)	2.4451*e* + 1 (1.05*e* + 0)	2.6761*e* + 1 (7.91*e* + 0)
		1000	4.4200*e* + 1 (7.77*e* + 0)	7.7862*e* + 1 (7.30*e* + 0)	7.9824*e* + 1 (2.29*e* + 0)	5.8910*e* + 1 (1.32*e* + 0)	5.0172*e* + 1 (1.07*e* + 0)

DTLZ3	5	500	4.3020*e* + 3 (1.57*e* + 3)	2.3235*e* + 4 (6.13*e* + 3)	1.2346*e* + 4 (7.98*e* + 0)	1.8602*e* + 4 (7.53*e* + 2)	3.1291*e* + 4 (6.31*e* + 2)
		1000	1.0670*e* + 4 (2.63*e* + 3)	4.3783*e* + 4 (8.98*e* + 3)	2.4844*e* + 4 (1.47*e* + 1)	6.0220*e* + 4 (1.81*e* + 3)	7.6232*e* + 4 (9.67*e* + 3)
	10	500	1.3306*e* + 4 (1.60*e* + 3)	4.1894*e* + 4 (2.65*e* + 3)	1.4213*e* + 4 (1.03*e* + 1)	3.8615*e* + 4 (7.37*e* + 2)	3.9350*e* + 4 (7.75*e* + 2)
		1000	2.5625*e* + 4 (4.16*e* + 3)	8.4954*e* + 4 (6.42*e* + 3)	2.7420*e* + 4 (9.93*e* + 0)	8.5528*e* + 4 (1.17*e* + 3)	8.8290*e* + 4 (1.15*e* + 3)

DTLZ4	5	500	2.6523*e* + 1 (1.65*e* + 0)	3.5059*e* + 1 (5.11*e* + 0)	2.6238*e* + 1 (3.03*e* + 0)	5.6372*e* + 0 (3.94*e* - 1)	5.9172*e* + 0 (7.12*e* - 1)
		1000	5.8758*e* + 1 (3.00*e* + 0)	6.6782*e* + 1 (1.20*e* + 1)	6.9454*e* + 1 (3.04*e* + 0)	2.2961*e* + 1 (9.24*e* − 1)	2.9794*e* + 1 (2.77*e* + 0)
	10	500	2.3290*e* + 1 (1.42*e* + 0)	3.3557*e* + 1 (9.70*e* + 0)	4.0018*e* + 1 (1.84*e* + 0)	2.4352*e* + 1 (8.56*e* − 1)	2.5171*e* + 1 (5.22*e* − 1)
		1000	4.9795*e* + 1 (3.90*e* + 0)	7.0596*e* + 1 (1.42*e* + 1)	8.0600*e* + 1 (2.05*e* + 0)	5.8910*e* + 1 (1.22*e* + 0)	5.6250*e* + 1 (1.07*e* + 0)

DTLZ5	5	500	2.8696*e* + 1 (1.44*e* + 0)	2.4279*e* + 1 (7.19*e* + 0)	3.5655*e* + 1 (1.04*e* + 0)	7.8308*e* + 0 (6.53*e* − 1)	2.9302*e* + 0 (2.18*e* − 1)
		1000	6.3241*e* + 1 (2.51*e* + 0)	3.7272*e* + 1 (1.12*e* + 1)	7.4941*e* + 1 (1.88*e* + 0)	2.7873*e* + 1 (1.40*e* + 0)	1.6365*e* + 1 (4.94*e* − 1)
	10	500	2.2663*e* + 1 (4.07*e* + 0)	2.2874*e* + 1 (7.97*e* + 0)	4.0439*e* + 1 (4.57*e* + 0)	2.7748*e* + 1 (1.08*e* + 0)	2.6317*e* + 1 (8.24*e* + 0)
		1000	4.8397*e* + 1 (7.02*e* + 0)	4.8756*e* + 1 (1.63*e* + 1)	8.1209*e* + 1 (2.71*e* + 0)	6.3840*e* + 1 (1.28*e* + 0)	4.9904*e* + 1 (1.13*e* + 0)

DTLZ6	5	500	8.8879*e* + 0 (1.39*e* + 0)	4.2732*e* + 2 (2.36*e* + 1)	9.4574*e* + 0 (8.51*e* + 0)	3.8495*e* + 2 (4.90*e* + 0)	3.6416*e* + 2 (2.58*e* + 0)
		1000	1.9706*e* + 1 (3.29*e* + 0)	8.9090*e* + 2 (2.39*e* + 1)	2.9199*e* + 1 (1.29*e* + 1)	8.1717*e* + 2 (6.01*e* + 0)	8.0078*e* + 2 (3.00*e* + 0)
	10	500	5.4188*e* + 1 (1.00*e* + 1)	4.2710*e* + 2 (1.53*e* + 1)	7.0212*e* + 1 (1.23*e* + 1)	4.1523*e* + 2 (2.44*e* + 0)	4.1207*e* + 2 (2.66*e* + 0)
		1000	1.0773*e* + 2 (3.16*e* + 1)	8.6234*e* + 2 (4.55*e* + 1)	1.1227*e* + 2 (8.94*e* + 1)	8.5524*e* + 2 (2.99*e* + 0)	8.5757*e* + 2 (2.39*e* + 0)

**Table 3:** Average rankings of the Friedman test.

Algorithm	Ranking
MALSMEA	2.1667
GLMO	3.4583
LCSA	3.6667
VaEA	2.9583
RVEA	2.75

**Table 4:** Comparison of running time between MALSMEA and the other four algorithms.

Algorithm	Time
MALSMEA	2.3113*e* + 2
GLMO	2.0182*e* + 2
LCSA	4.3017*e* + 1
VaEA	1.2587*e* + 2
RVEA	6.8803*e* + 1

**Table 5:** Performance comparison between MALSMEA and four algorithms with respect to the average IGD values on the LSMOP1–LSMOP9 (gray values represent the best values in each row).

Problem	*m*	*D*	MALSMEA	GLMO	LCSA	VaEA	RVEA
LSMOP1	5	500	1.3173*e* + 0 (1.55*e* − 1)	9.9913 *e* − 1 (1.05*e* − 1)	9.3999*e* − 1 (5.30*e* − 3)	1.6687*e* + 0 (2.66*e* − 1)	1.2713*e* + 0 (1.54*e* − 1)
		1000	1.3109*e* + 0 (1.61*e* − 1)	1.2099*e* + 0 (5.21*e* − 1)	9.3942*e* − 1 (2.67*e* − 3)	3.6704*e* + 0 (4.00*e* − 1)	2.6898*e* + 0 (2.09*e* − 1)
	10	500	1.2008*e* + 0 (1.89*e* − 1)	5.9934*e* + 0 (2.79*e* + 0)	1.2010*e* + 0 (1.16*e* − 3)	4.1745*e* + 0 (1.28*e* + 0)	1.6742*e* + 0 (3.51*e* − 1)
		1000	1.1728*e* + 0 (1.53*e* − 1)	7.9449*e* + 0 (3.19*e* + 0)	1.1938*e* + 0 (2.75*e* − 3)	7.0153*e* + 0 (6.50*e* − 1)	4.0353*e* + 0 (9.27*e* − 1)

LSMOP2	5	500	1.5237*e* − 1 (1.77*e* − 3)	1.8423*e* − 1 (5.16*e* − 3)	1.9821*e* - 1 (6.56*e* − 3)	1.6390*e* - 1 (1.71*e* − 3)	1.6594*e* − 1 (9.99*e* − 4)
		1000	1.3444*e* − 1 (1.08*e* − 3)	1.6139*e* − 1 (4.75*e* − 3)	1.7402*e* − 1 (3.87*e* − 3)	1.4188*e* − 1 (1.73*e* − 3)	1.4299*e* − 1 (8.72*e* − 4)
	10	500	2.8094*e* − 1 (6.69*e* − 3)	3.3525*e* − 1 (7.25*e* − 3)	3.6322*e* − 1 (8.55*e* − 3)	3.1995*e* − 1 (3.89*e* − 3)	2.8197*e* − 1 (3.56*e* − 3)
		1000	2.3979*e* − 1 (2.71*e* − 3)	2.8301*e* − 1 (5.22*e* − 3)	3.0751*e* − 1 (7.90*e* − 3)	2.6900*e* − 1 (1.85*e* − 3)	2.3980*e* − 1 (3.04*e* − 3)

LSMOP3	5	500	1.1955*e* + 1 (3.86*e* + 0)	1.3626*e* + 0 (6.23*e* − 1)	9.5883*e* − 1 (0.00*e* + 0)	1.6636*e* + 1 (4.85*e* + 0)	4.7605*e* + 0 (1.27*e* + 0)
		1000	1.3419*e* + 1 (4.38*e* + 0)	1.4773*e* + 0 (5.34*e* − 1)	9.5883*e* − 1 (0.00*e* + 0)	1.6875*e* + 1 (5.62*e* + 0)	8.7885*e* + 0 (1.03*e* + 0)
	10	500	1.2546*e* + 1 (1.59*e* + 0)	2.1075*e* + 2 (3.43*e* + 2)	1.8733*e* + 0 (1.57*e* − 3)	1.7999*e* + 1 (3.05*e* + 0)	2.4510*e* + 0 (4.99*e* − 1)
		1000	1.3071*e* + 1 (1.29*e* + 0)	1.1423*e* + 4 (1.26*e* + 2)	1.9179*e* + 0 (8.35*e* − 4)	1.9379*e* + 1 (2.80*e* + 0)	4.3816*e* + 1(1.40*e* + 0)

LSMOP4	5	500	2.8356*e* - 1 (8.13*e* - 3)	3.3698*e* − 1 (1.31*e* − 2)	3.2856*e* − 1 (9.98*e* − 3)	3.0856–1 (5.78*e* − 3)	2.8894*e* − 1 (2.96*e* − 3)
		1000	2.1150*e* - 1 (5.31*e* − 3)	2.4674*e* − 1 (7.40*e* − 3)	2.5458*e* − 1 (6.51*e* − 3)	2.1842*e* − 1 (3.10*e* − 3)	2.1661*e* − 1 (1.51*e* − 3)
	10	500	3.3748*e* − 1 (5.61*e* − 3)	3.9190*e* − 1 (1.04*e* − 2)	4.3146*e* − 1 (1.52*e* − 2)	3.7828*e* − 1 (3.79*e* − 3)	3.4044*e* − 1 (3.98*e* − 3)
		1000	2.7003*e* − 1 (2.36*e* − 3)	3.1838*e* - 1 (8.76*e* − 3)	3.5483*e* − 1 (6.41*e* − 3)	3.0457*e* − 1 (3.65*e* − 3)	2.7902*e* − 1 (3.82*e* − 3)

LSMOP5	5	500	4.5817*e* − 1 (5.45*e* − 3)	3.3566*e* + 0 (3.16*e* + 0)	4.6074*e* − 1 (3.81*e* − 2)	4.5633*e* + 0 (3.26*e* − 1)	1.8603*e* + 0 (3.83*e* − 1)
		1000	4.5647*e* − 1 (2.97*e* − 2)	8.3782*e* + 0 (6.28*e* + 0)	4.5874*e* − 1 (1.99*e* − 2)	7.4372*e* + 0 (7.67*e* − 1)	3.3211*e* + 0 (5.08*e* − 1)
	10	500	6.5504*e* − 1 (4.37*e* − 2)	1.6148*e* + 1 (8.45*e* + 0)	1.1132*e* + 0 (8.69*e* − 2)	8.4930*e* + 0 (1.21*e* + 0)	3.0758*e* + 0 (5.69*e* − 1)
		1000	6.6973*e* − 1 (6.22*e* − 2)	1.4246*e* + 1 (6.02*e* + 0)	1.1087*e* + 0 (9.32*e* − 2)	1.0274*e* + 1 (1.04*e* + 0)	6.1324*e* + 0 (5.95*e* − 1)

LSMOP6	5	500	1.2094 *e*+ 0 (1.33*e* − 1)	5.3807*e* + 2 (1.68*e* + 3)	1.2106*e* + 0 (3.67*e* − 2)	1.1135*e* + 1 (5.75*e* + 0)	8.3040*e* + 0 (1.66*e* + 1)
		1000	1.2188*e* + 0 (8.52*e* − 2)	2.5183*e* + 3 (4.35*e* + 3)	1.2549*e* + 0 (5.34*e* − 2)	1.4415*e* + 2 (3.65*e* + 1)	5.3053*e* + 1 (2.99*e* + 1)
	10	500	1.4348*e* + 0 (1.42*e* − 1)	6.0471*e* + 1 (1.88*e* + 2)	1.4179*e* + 0 (8.13*e* − 2)	1.3763*e* + 2 (2.90*e* + 2)	1.2580*e* + 0 (1.09*e* − 1)
		1000	1.4961*e* + 0 (1.46*e* − 1)	7.6272*e* + 2 (3.01*e* + 3)	1.3573*e* + 0(7.95*e* − 2)	1.5136*e* + 0 (8.68*e* − 3)	1.2743*e* + 0 (9.00*e* − 2)

LSMOP7	5	500	1.3323*e* + 0 (6.63*e* − 2)	2.4841*e* + 0 (3.00*e* − 1)	1.0912*e* + 0 (1.46*e* − 2)	2.9317*e* + 0 (1.47*e* − 1)	1.2645*e* + 0 (1.88*e* − 1)
		1000	1.3577*e* + 0 (6.26*e* − 2)	1.7911*e* + 0 (1.01*e* − 1)	1.0321*e* + 0 (1.40*e* − 2)	1.9182*e* + 0 (5.40*e* − 2)	1.1214*e* + 0 (8.68*e* − 2)
	10	500	1.3995*e* + 0 (7.97*e* − 2)	3.5137*e* + 4 (1.36*e* + 4)	1.5578*e* + 0 (5.12*e* − 2)	1.0739*e* + 3 (7.45*e* + 2)	2.6040*e* + 1 (6.95*e* + 0)
		1000	1.4663*e* + 0 (1.11*e* − 1)	3.7805*e* + 4 (1.15*e* + 4)	1.5933*e* + 0 (5.63*e* − 2)	2.7102*e* + 3 (1.09*e* + 3)	1.4501*e* + 2 (3.01*e* + 1)

LSMOP8	5	500	3.8850*e* − 1 (2.43*e* − 2)	1.1661*e* + 0 (7.11*e* − 2)	3.8922*e* − 1 (1.02*e* − 2)	1.1767*e* + 0 (9.67*e* − 3)	9.3066*e* - 1 (1.19*e* − 1)
		1000	3.9206*e* − 1 (3.27*e* − 2)	1.0697*e* + 0 (9.46*e* − 2)	3.9962*e* − 1 (8.72*e* − 3)	1.1544*e* + 0 (1.25*e* − 3)	8.9791*e* - 1 (1.45*e* − 1)
	10	500	6.4152*e* − 1 (4.00*e* − 2)	1.2619*e* + 1 (4.49*e* + 0)	9.6995*e* − 1 (9.27*e* − 2)	2.8446*e* + 0 (5.01*e* − 1)	1.4025*e* + 0 (1.12*e* − 1)
		1000	6.2434*e* − 1 (3.37*e* − 2)	1.1402*e* + 1 (4.19*e* + 0)	1.0886*e* + 0 (1.06*e* − 1)	4.0270*e* + 0 (6.02*e* −1)	2.6957*e* + 0 (3.85*e* − 1)

LSMOP9	5	500	2.8005*e* + 0 (2.91*e* − 8)	2.9775*e* + 0 (9.23*e* − 2)	2.9985*e* + 0 (8.77*e* − 3)	1.2971*e* + 1 (2.27*e* + 0)	2.5483*e* + 1 (6.20*e* + 0)
		1000	2.9801*e* + 0 (9.11*e* − 2)	2.9976*e* + 0 (9.44*e* − 2)	3.0005*e* + 0 (0.00*e* + 0)	3.5883*e* + 1 (3.99*e* + 0)	5.5544*e* + 1 (1.95*e* + 1)
	10	500	6.4182*e* + 0 (1.93–1)	6.5037*e* + 0 (7.63*e* − 1)	6.5321*e* + 0 (3.65*e* − 15)	3.6094*e* + 2 (2.89*e* + 1)	2.7313*e* + 2 (9.11*e* + 1)
		1000	6.3652*e* + 0 (2.05*e* − 1)	6.3891*e* + 0 (1.06*e* + 0)	6.5321*e* + 0 (3.65*e* − 15)	5.0223*e* + 2 (2.77*e* + 1)	3.4370*e* + 2 (9.37*e* + 1)

**Table 6:** HV values and optimized results of four algorithms (values in bold represent better results).

Dataset	Algorithm	HV	Feature	Accuracy	Relevance	Redundancy	Interclass distance	Intraclass distance
Heart	MALSMEA	**0.9972**	**6**	**0.7979**	0.4615	0.1923	**0.0802**	**0.0123**
	W-MOSS	0.9962	7	0.7667	0.5385	0.2692	0.0769	0.0128
	W-QEISS	0.9943	8	0.7604	**0.6154**	0.3590	0.0764	0.0130
	F-QEISS	0.9980	7	0.7811	0.5385	**0.0256**	0.0798	0.0125

Zoo	MALSMEA	**0.9979**	**5**	**0.9842**	0.3125	0.0833	**0.0637**	**0.0074**
	W-MOSS	0.9975	7	0.9816	0.4375	0.1750	0.0622	0.0085
	W-QEISS	0.9972	7	0.9697	**0.5000**	0.2333	0.0615	0.0076
	F-QEISS	0.9977	6	0.9556	0.3750	**0.0167**	0.0609	0.0083

Iris	MALSMEA	**0.9351**	**2**	**0.9387**	0.5000	**0.1667**	**0.2574**	**0.1667**
	W-MOSS	0.9234	3	0.9071	**0.7500**	0.5000	0.2566	0.1673
	W-QEISS	0.9236	3	0.9049	0.5655	0.1765	0.2571	0.1670
	F-QEISS	0.9247	3	0.9187	**0.7500**	**0.1667**	0.2569	0.1668

Musk1	MALSMEA	**0.9697**	**11**	**0.6173**	0.0663	**0.0045**	**7.3102*e* − 5**	**0.0060**
	W-MOSS	0.9693	12	0.6130	0.0723	0.0048	7.3023*e* − 5	**0.0060**
	W-QEISS	0.9603	13	0.5956	**0.0783**	0.0057	7.3037*e* − 5	0.0067
	F-QEISS	0.9627	13	0.6069	**0.0783**	0.0057	7.3026*e* − 5	0.0062

## Data Availability

The details of the four UCI datasets utilized are shown in Table 1.
